# Visual Novels in Maxillofacial Surgery Education: A Hidden Gem

**DOI:** 10.7759/cureus.43125

**Published:** 2023-08-08

**Authors:** Muslat A Bin Rubaia’an

**Affiliations:** 1 College of Medicine, King Saud University, Riyadh, SAU; 2 College of Dentistry, Riyadh Elm University, Riyadh, SAU

**Keywords:** style, approach, teacher, student, learning

## Abstract

Visual novels are vital learning tools that promote student satisfaction and help them acquire the skills and knowledge needed to solve problems. They use interactive narrative designs to create a list of effective teaching strategies to enhance user experience. The practical use of these resources would require orienting learners to online training to improve their understanding of content. Educators must recognize the uniqueness of online learning designs and tools to develop effective strategies to enhance user understanding and improve student-teacher interactions. Visual novels' effectiveness in maxillofacial surgery education should involve assessing the play and diverting attention from lesson content to how effectively students learn. Deduction activities and features in the game should be informative and entertaining and make considerations for short- and long-term objectives like delivering feedback on immediate and appropriate tool use and communication.

Visual novels are interactive, allowing users to control story development through specific actions. These interactive narratives are primarily used in academic and industrial contexts covering diverse subjects. These models have become increasingly crucial in maxillofacial surgery, providing technical help and improving surgery's quality and speed. Instructors could develop the games as a course component or integrate them with other course materials because they are easy to access and download. However, some visual novels have failed to incorporate educational content delivery with interactive experiences. This indicates a research gap in interactive narrative designs and the primary content delivery mechanisms. Incorporating these resources into education and practice would reduce reliance on experience alone, reducing surgical errors and mistakes. This review highlights the importance of visual novels in maxillofacial surgery education and emphasizes their educational role.

## Introduction and background

The integration of digital technologies into dental curricula has been implemented with the aim of augmenting students' spatial ability, interaction, analytical skills, and clinical correlations across various dental disciplines [1]. To facilitate the transfer of knowledge from theory to practice to today’s students, particularly the Millennials, it is imperative to advocate for a paradigm shift within the dental faculty and equip instructors with e-teaching training [1]. The global pandemic of COVID-19 has underscored the necessity of exploring alternative educational avenues, such as E-learning modules, virtual conferencing, surgical skills simulation technologies, and serious games [2,3]. A serious game is defined as an interactive application, which may or may not have a significant hardware component, that possesses a challenging objective, provides an enjoyable and engaging experience, includes a scoring system, and imparts the user with skills, knowledge, or perspectives that are beneficial in real-life situations [4]. Educators should substitute non-interactive texts and reading resources with serious games, as they are powerful pedagogical tools that offer comparable educational results to conventional methods and in a more engaging manner [3].

Technology-based education has become increasingly important in diverse areas, such as maxillofacial surgery, as it provides technical assistance and enhances the surgery's quality and rapidity [5,6]. Past years have witnessed the increased use and importance of technology, which has proven crucial in medical training and surgical interventions [6,7]. These resources provide vital information regarding real environments, such as visuals, helping to improve comprehension [8]. Serious games enable professionals to engage in multitasking roles by training them to handle multiple cases simultaneously [4]. The significance of non-technical skills in reducing medical errors within dynamic high-risk settings, such as the operating room or the emergency department, has been widely acknowledged [4]. Serious games provide knowledge that is represented in an interactive visual format, enabling students to learn about the surgical field.

Maxillofacial surgeons perform various procedures, like making incisions on facial soft tissues. The incisions are delicate procedures made from one layer to another to prevent injury to blood vessels and nerves. Hence, these procedures need extensive training. The traditional training approaches mainly involve observing or shadowing in the operation rooms [9]. Trainees then gradually begin operating on patients under the supervision of experienced surgeons [7]. However, students may encounter difficulties obtaining opportunities for early exposure to surgical practices [9]. This aspect makes it increasingly challenging to master surgical skills due to the extensive training durations required, scarce resources, and limited opportunities [10]. Technology developments in virtual reality and serious games have facilitated the use of advanced techniques in maxillofacial surgery education to overcome these challenges [11]. Such developments have also allowed for the practice of advanced procedures, such as bilateral sagittal osteotomy, Le Fort 1 osteotomy, and condylectomy of the temporomandibular joint [11]. Virtual reality and serious games have proven effective at improving students' knowledge and teaching clinical reasoning and patient evaluation [11].

Visual novels, a genre primarily originating from Japan, are narrative-driven serious games that significantly emphasize player choices [12]. In recent times, there has been a notable increase in the popularity of visual novels in education [12]. Visual novels are effective teaching tools as they comprise adequate plans for enactment, feedback, and rewards to facilitate learning [12]. They ensure that learning activities and processes are informative and entertaining. Visual novels are easy to use and would help in engaging large audiences [13]. These learning resources would help evaluate specific skills before beginning instruction and track development throughout learning. They provide learners with virtual contexts for testing their knowledge and ideas safely. They would help to make learning more enjoyable and engaging to motivate students to internalize knowledge [12].

Despite the potential benefits, visual novels are underutilized in the field of maxillofacial surgery education. Using visual novels in maxillofacial surgery training would be vital, as it would ensure that trainees acquire high knowledge and dexterity to ensure correct and smooth practice [10]. In addition, visual novels would provide learners with secure and reusable virtual models that increase their knowledge by engaging them in an interactive practice context. This paper aimed to provide a brief review of the literature to shed light on visual novels and to emphasize the educational role that visual novels would play in maxillofacial surgery training.

## Review

Development of visual novels

While there is no universally accepted definition of a visual novel, Cavallaro gives us a good idea of what they are: (1) narratively driven experiences that are primarily text, backgrounds, and dialogue boxes with character sprites; (2) illustrations/graphics that are presented to the player at critical points in the game narratives; and (3) a branching narrative with multiple endings, depending on the player's choices [14]. Visual novels are digital applications with a graphical user interface that showcase multiple layers of artwork to create a composition. These features enable character art and background images to appear onscreen and disappear as the story continues [15]. Visual novels differ from graphic novels, a class of resources not digital or applications [16]. Graphic novels use a series of static comic book panels that do not provide interactivity or animation unless it is a webcomic. However, both resources combine visual storytelling with some texts and artworks. Visual novels are commonly used for entertainment but are increasingly evaluated as an approach to delivering information and knowledge [17]. The resource is often used in public health education to enable patients to manage their hospital stays and care experiences [18]. Visual novels could be used as more interactive media to attain objectives similar to those in graphic medicine. Visual novels are designed with the understanding that knowledge is interactive, showing the need to promote communication and information exchange [19]. These resources present multiple tasks to be performed in a three-dimensional world, including chat features for socializing. This aspect takes learning to a simulated level and increases the enjoyability of educational processes.

Visual novels have wide educational implications [13]. Since the 1970s, educational computer games have been used, but in recent years, this subject has drawn much more attention [20,21]. Many students prefer this learning method over traditional classroom lectures because it allows young people to use their interest in games in their academic learning processes [22]. Despite their increased recognition as powerful learning tools, numerous educators and policy-makers may perceive new technological approaches as a distraction from academic learning, civic engagement, and future opportunities. However, evidence has shown visual novels would promote connected learning and help cross the gap between in- and out-of-school learning [23]. This aspect would also help to address new equity gaps from privatizing learning. Visual novels would provide opportunities for connecting education provided using virtual technology across diverse settings.

Visual novels are commonly used as educational resources for health specialties [24-26]. Visual novels could cover various subjects, from fictional languages to self-efficacy in coding [12]. These resources are popular within educational contexts as they are easy to use and focus on storytelling and role-playing [13]. They are crucial for engaging large audiences and providing users with various development tools such as Novelty, Unity, and Ren'Py [13]. These supports make developing educational game designers with diverse technical skills and expertise increasingly easy. Non-interactive texts and reading materials have been substituted with visual novels by educators [13]. They validate their decisions with arguments from studies showing visual novels are powerful pedagogical tools [13,27-29]. Visual novels are vital tools and resources in different areas of medical education.

The preparation of a script and the addition of scenes, characters, dialogue, and choices, as well as the selection of settings and characters, are typical steps in the workflow using the Visual Novel Maker engine (Degica 2019). The games may be implemented by teachers as a component of a course and integrated with other course materials, or they may be exported as easily accessible web content or downloadable files. Visual novel games can be easily downloaded from diverse platforms and played on smartphones or computers. The ease of access is reinforced by visual appeal, which motivates users to play continuously [13]. These novel games comprise various graphic elements, including characters, narration, and styles [30]. The game design phase, game software design phase, game implementation and publishing phase, and game-based learning and feedback phase comprise the visual novel life cycle [30]. Several stages compose each phase. Stages are given names based on the processes they represent, such as the stages of game idea generation, architectural design, and programming [30]. Each stage includes a process and a work product, such as the requirements development stage, which results in the requirements specification document as a work product [31].

Visual novels benefit the academic sector, but the resources have been under-researched, especially considering their educational effectiveness [13]. Numerous educational visual novels have been ineffective at combining educational content delivery and interactive narrative experiences [32]. This issue results from a research gap in interactive narrative designs and the underlying mechanisms for delivering educational content. The games would be practical tools not only for entertainment but also for learning. Therefore, educators must develop attractive learning designs to increase learners' interest.

Adequate research would help determine the most effective design elements or story content compatible with the learning process [13]. It would help address issues such as content that is difficult to understand, which results in extraneous cognitive load or distracts players from learning. Design choices could be aligned with educational goals by ensuring that content delivery is directly integrated into the visual novel's underlying mechanisms and elements [33]. This aspect would help generate practical educational game designs and eliminate unnecessary confusion for players. Creating a taxonomy could address the research gap and ambiguity concerning the interaction design and mechanisms promoting content delivery [13]. The tool would provide a common language for designers and researchers to exchange knowledge and develop new classification systems, structures, prototypes, and research questions [13].

Rationale

Visual novels are popular in education, where they are used to cover different topics and learning objectives. These tools are popular in the sector because they provide learners with increased accessibility to diverse genres. They are also easy to implement as they use narrative approaches and role-playing in various ways to enhance learning [34]. Educational games are framing devices and tools for contextualizing and motivating learning [35]. Visual novels are valuable tools in undergraduate science education. They would help enhance the instructional approaches that focus on teaching subject matter rather than science processes and skills. These tools promote science process skills, including observing, hypothesis testing, communication, collaboration, and problem-solving [23]. These are among the core skills of scientific training but are rarely taught in undergraduate education. Educators often perceive the skills as too challenging or time-consuming to train, influencing them to prefer teaching other subjects. Visual novels would address these issues by increasing students' ability to make observations, analyze data, perform a literature search, and develop a hypothesis. These learning tools would give students vital skills that would help them in other science courses and contexts.

Visual novels are practical for learning as they promote student satisfaction. Learners often find working with these resources that increase their interaction with usual scientific subjects exciting [36]. Visual novels provide learners with more engaging fictional scenarios and media that increase their enjoyment and engagement in learning. These resources would enable students to realize their growth by using the acquired skills and knowledge to solve problems [23]. Visual novels also give learners increased flexibility as they can complete activities creatively. They enable students to use their imagination and creativity in investigating new areas and gain knowledge that would benefit the discipline and society. The visual tools also improve student collaboration as they require learners to work in groups where they share and discuss their hypotheses [37]. This aspect enables students to practice and improve their communication skills while critically evaluating each other's ideas.

Educational principles behind visual novel design

**Figure 1:**
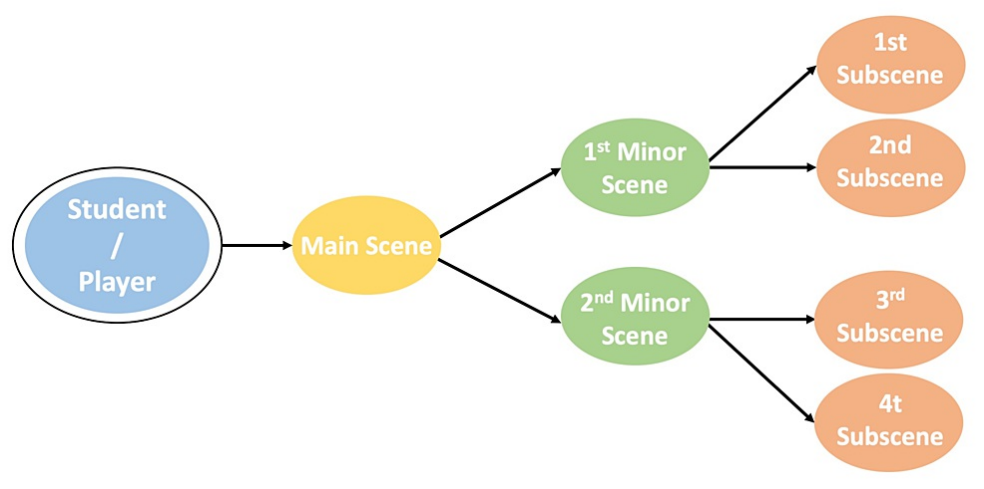
Different visual novel routes. The author designed this figure, which was adopted with permission from the original creator (Garcia MB) [38].

Visual novels use the interactive narrative design to help develop a taxonomy of teaching strategies for improving the user experience. These methods would enhance the delivery of educational content and its integration with the story. Interactive narratives are structured using graph theory concepts and descriptions that often indicate the player's influence in the story's progression. Various theories and taxonomies have been suggested for evaluating the interactive narrative structure, most using graph-based representations. The taxonomies promote different kinds of advancement, including progress through discovery [13]. This refers to the processes players must use to find story content, such as the character's life and choices. Progression by choice enables players to move to another level of the story, where they begin making direct decisions, such as dialogue choices. Progression through in-game systems occurs when players interact with narrative structures such as combat or resource management. Progression through scripted scenarios occurs when players perform specific actions to progress through the story, such as answering simple questions correctly. Each progression choice will result in a different novel route (Figure 1) [38].

There is a need for close-reading strategies to discover patterns to guide the development of principles and standards [13]. Another technique would involve using mechanics, dynamics, and aesthetics frameworks to describe and evaluate educational visual novels in a high-level manner. This approach would help to connect specific gameplay elements of educational visual novels to teaching strategies. Most definitions contend that visual novels are interactive and narrative-focused with anime influences. They have proven effective at teaching educational content better than traditional interactive narratives. Recent studies have shown that educational visual novels enhance students' self-efficacy and knowledge.

Developing a taxonomy of teaching strategies for visual novels requires an adequate understanding of the interactive narrative design and its relationship to the user experience [13]. Understanding the delivery approaches for educational content and how they can be effectively integrated into the story is especially important. Current initiatives to capture and describe game design have generated languages and computational frameworks. However, it is vital to increasingly focus on the interaction design and the interactive narrative's presentation from the learners' perspective [13]. Virtual reality in maxillofacial surgery training has proven to enhance knowledge and skills, as students can choose specific applications and operations from the surgical menu. This would ensure they understand and select the appropriate tools for diverse surgical procedures.

How visual novels should be used in maxillofacial surgery education

The degree of self-confidence that surgeons possess affects their performance, level of professional satisfaction, and prospects for future success [39]. The absence of confidence among trainees with limited experience can result in inadvertent errors during surgical procedures. The field of maxillofacial surgery currently faces a deficiency in educational and assessment resources aimed at enhancing the self-confidence of surgical residents [39]. Hence, there is a necessity to implement innovative educational resources. Game-based education has the potential to offer a solution to this obstacle.

Games and learning are vital for engaging students and providing pleasurable experiences. This aspect could help explain why educational games are still entrenched in the digital culture. Therefore, learning institutions must comprehend the complexities of integrating virtual educational resources to cope with and survive in a technologically biased pedagogical landscape [38]. Gaming approaches in education will determine the investment students make in learning. Technology would increase students' curiosity about the content. This aspect would influence them to complete more tasks and operations, improving their education. Game-based education offers numerous advantages, such as its suitability for adult learners and the potential to facilitate experiential and repetitive education [40]. In addition, the active involvement of learners contributes to the acquisition of knowledge, the development of attitudes and skills, the customization of learning experiences, and the enhancement of the learning process and outcomes [40]. Considering the various benefits that have been discussed, it is essential to acknowledge that integrating games in educational settings is not without limitations. The utilization of gamification techniques in educational settings has been found to result in a reduction in learners' attention spans [41]. In addition, some learners may perceive games as threatening and intimidating due to their competitive nature [40]. Due to its ease of access and availability online, junior trainees may practice advanced surgical procedures with no supervision. Even worse, unlicensed laypersons might perform these procedures. Additionally, students may encounter cyber-security threats, such as unauthorized access to their personal information [42]. Students may also face identity threats, which involve the misuse of their identities as users [42]. Thus, serious games from unknown sources should be used with extreme caution.﻿ Cost is another major limitation. The development of a game requires significant costs due to the need for additional personnel with expertise in coding since the educators might have no programming backgrounds or have limited available time due to academic or clinical obligations. Moreover, additional costs are needed for its additional resources, such as animations, graphics, and music [41].

**Figure 2:**
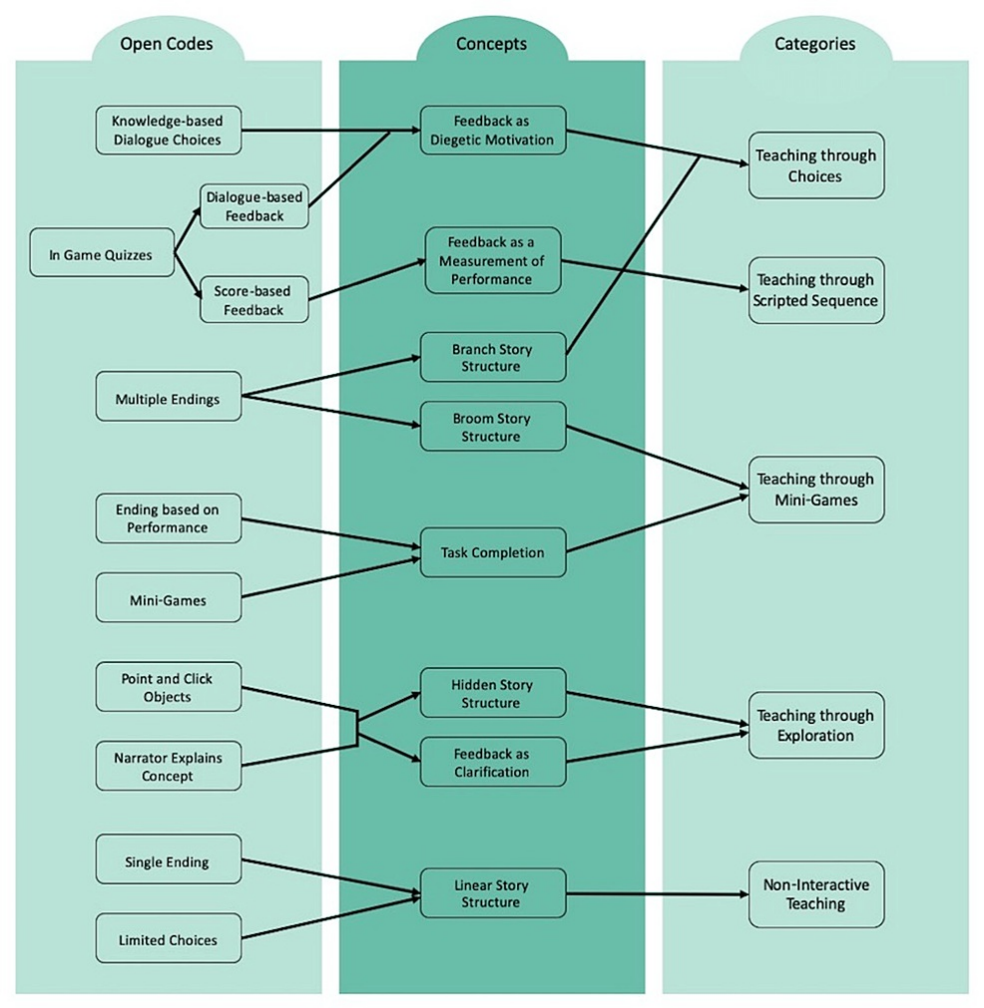
The overall process from coding to concepts to categories. This figure, which the author designed, was adopted from " A (Visual) Novel Route to Learning: A Taxonomy of Teaching Strategies in Visual Novels" (https://doi.org/10.1145/3402942.3403004) by Camingue et al. (https://dl.acm.org/profile/99659579276), and used under CC BY 4.0 (https://creativecommons.org/licenses/by/4.0/).

Developing a serious game, such as a visual novel, is a complicated process that necessitates the collaboration of a diverse group of professionals, including educators, game designers, programmers, illustrators, and occasionally actors [43]. Educators play a crucial role in verifying the accuracy of the content and ensuring that the game adheres to pedagogical principles, making it appropriate for educational purposes. They are involved in the user-centered design phases, as they are the only team members who interact with the intended target audience [43]. Moreover, educators should ensure proper and adequate information communication to effectively develop interactive narratives, especially those used for framing in education and visual novels [44]. Learning content should be effectively communicated across diegetic boundaries. However, there is a shortage of fully developed and generalizable diegesis models, especially in digital games. Despite these factors, Camingue et al. explored a proposed framework using a player-centered approach to evaluating diegesis [13]. The strategy focuses on how information is presented and how players interact with the game. The process promotes the conceptualization of diegesis as the story-world's frame, confining the story within the window in which the interactive narrative is represented. The process of thematic analysis for the taxonomy of teaching strategies in visual novels includes familiarizing oneself with the data and coding of narrative progression mechanics. It also involves developing initial themes to identify concepts using the story's structure, player interaction, and how diegetic feedback is presented. The themes are then reviewed to determine taxonomy categories and analyzed to report how educational content is delivered through teaching strategies (Figure 2) [13].

The practical application of visual novels would require orienting learners for online training to ensure they understand the delivered content [45]. It is crucial to consider the ease of generating content alongside the multiple features to include when designing educational games. Adopting a framework with a collaboration mode is vital, as it would help users interact and contribute to creating games. This aspect is crucial to avoid confusing students due to previous interactions in other online learning contexts [30]. Therefore, students must acclimate to the new e-learning and training context because of the unique delivery method, as it uses game mechanisms [46]. Learners may find it challenging to understand what to do with the online learning resource due to the difficulty and unfamiliarity of its navigation compared to its face-to-face counterpart [47]. Educators must recognize that online learning designs and methodologies are unique and ensure that users have an adequate basic understanding of operations to enhance student-teacher interactions.

Assessment

Past decades have witnessed increased research and interest in developing tools for supporting education. Various products have been designed, including pervasive games and visual novels, that have proven to be potent mediators for learning. This is because of their interactive, immersive, personalized, and knowledge-oriented features that improve understanding [38]. One of the approaches to assessing the effectiveness of such tools is the duration taken to absorb content or the time on task. Visual novels are crucial tools as they stimulate cognitive processes such as reading, inference-making, deductive and inductive reasoning, and problem-solving. The resources can also motivate users to spend more time on activities, directly influencing learning and skill development [48].

The effectiveness of visual novels in teaching should not be evaluated in blocks. Assessments should assess the play, diverting attention from lesson content to how well students learn. The game must exhibit adequate plans for enactment, feedback, and reward to promote learning [49]. Such games' deductions, activities, and features should be informative and entertaining. The procedures should make adequate considerations for short and long-term goals, such as providing feedback on immediate and appropriate tool use and communication [19]. They should also offer rewards or stimuli for exhibiting desired behaviors or attaining the targeted skills. Evaluating the skills before initiating the instruction and tracking their gradual development throughout the learning activity is crucial. This approach would provide instructors with vital knowledge to determine the effectiveness of their teaching approaches and measures to streamline their instruction.

Past years have witnessed the development of various visual novels for health science students [23]. The student responses from the written reflection indicate that this particular form of education effectively delivered educational advantages. It effectively involved students in developing cognitive skills related to acquiring and applying knowledge, all while providing a pleasurable experience [23]. The potential of visual novels to facilitate active participation and knowledge acquisition within the field of maxillofacial surgery training is evident. Incorporating visual novels into maxillofacial surgery education and practice would help reduce surgical errors and mistakes from relying on experience alone. Visual novels would provide students or trainees with a safe virtual context to test their knowledge and ideas, enabling them to conduct more accurate surgeries. Future research should seek knowledge regarding the implementation of visual novels in maxillofacial surgery education.

## Conclusions

Visual novels are crucial resources and frameworks in various areas of medical education, including maxillofacial surgery. They are effective for teaching and learning, providing users with virtual contexts to safely test their knowledge and ideas. Visual novels could be used to teach various subjects and are popular in educational settings as they are easy to use and focus on storytelling and role-playing. They make the learning process increasingly enjoyable, helping to engage and motivate students to internalize knowledge. Visual novels are crucial tools in diverse fields, such as maxillofacial surgery. They would help trainees gain knowledge and confidence and improve the quality and rapidity of their services. However, they may result in a reduction in their attention spans.

Educators could use visual novels separately or combine them with other course materials because they are easy to access. Educators should substitute non-interactive texts and reading resources with visual novels, as they are powerful pedagogical tools with elaborate plans for enactment, feedback, and rewards to facilitate learning.
